# Causal risk factor discovery for severe acute kidney injury using electronic health records

**DOI:** 10.1186/s12911-018-0597-7

**Published:** 2018-03-22

**Authors:** Weiqi Chen, Yong Hu, Xiangzhou Zhang, Lijuan Wu, Kang Liu, Jianqin He, Zilin Tang, Xing Song, Lemuel R. Waitman, Mei Liu

**Affiliations:** 1 Big Data Decision Institute (BDDI), Jinan University, Tianhe, Guangzhou, 510632 China; 2 Guangdong Engineering Technology Research Center for Big Data Precision Healthcare, Tianhe, Guangzhou, 510632 China; 3 Department of Internal Medicine, Division of Medical Informatics, University of Kansas Medical Center, Kansas City, Kansas, USA

**Keywords:** Acute kidney injury (AKI), Causality discovery, Causal feature selection, Machine learning, Predictive modeling, Dimension reduction

## Abstract

**Background:**

Acute kidney injury (AKI), characterized by abrupt deterioration of renal function, is a common clinical event among hospitalized patients and it is associated with high morbidity and mortality. AKI is defined in three stages with stage-3 being the most severe phase which is irreversible. It is important to effectively discover the true risk factors in order to identify high-risk AKI patients and allow better targeting of tailored interventions. However, Stage-3 AKI patients are very rare (only 0.2% of AKI patients) with a large scale of features available in EHR (1917 potential risk features), yielding a scenario unfeasible for any correlation-based feature selection or modeling method. This study aims to discover the key factors and improve the detection of Stage-3 AKI.

**Methods:**

A causal discovery method (McDSL) is adopted for causal discovery to infer true causal relationship between information buried in EHR (such as medication, diagnosis, laboratory tests, comorbidities and etc.) and Stage-3 AKI risk. The research approach comprised two major phases: data collection, and causal discovery. The first phase is propose to collect the data from HER (includes 358 encounters and 891 risk factors). Finally, McDSL is employed to discover the causal risk factors of Stage-3 AKI, and five well-known machine learning models are built for predicting Stage-3 AKI with 10-fold cross-validation (predictive accuracy were measured by AUC, precision, recall and F-score).

**Results:**

McDSL is useful for further research of EHR. It is able to discover four causal features, all selected features are medications that are modifiable. The latest research of machine learning is employed to compare the performance of prediction, and the experimental result has verified the selected features are pivotal.

**Conclusions:**

The features selected by McDSL, which enable us to achieve significant dimension reduction without sacrificing prediction accuracy, suggesting potential clinical use such as helping physicians develop better prevention and treatment strategies.

## Background

Acute Kidney Injury (AKI) is a common and highly lethal health problem, affecting 10–15% of all hospitalized patients and more than 50% of the intensive care unit (ICU) patients. Previous studies have found that an increase in serum creatinine (SCr) greater than 0.5 mg/dl was associated with a 6.5-fold increase in the odds of death, a 3.5-days increase in length of stay, and nearly $7500 hospital costs in excess [1, 2]. In accordance with Kidney Disease Improving Global Outcomes (KDIGO) criteria, AKI is staged into three phases with ascending severity and treatment complexity. Stage-3 AKI, in particular, is the most critical stage which is not only irreversible but would result in worse mortality rate. Early prediction of potential AKI, especially Stage-3 AKI, can help with early identification of the high-risk patients and thus allow more appropriate allocation of limited clinical resources [3]. In recent years, scholars have focused on the development of machine learning methods to facilitate early detection, diagnosis and intervention, helping clinicians to provide more suitable and timely management for patients at high risk for AKI, resulting in improved clinical outcomes. It has been argued that better use of electronic health records (EHR) is the key to realize this objective [4, 5].

However, Stage-3 AKI patients are very rare but with abundant features recorded. The employed EHR shows that only 179 (0.2%) patients acquired Stage-3 AKI out of 89, 685 patients over the past 10 years, while hundreds even thousands of features have been observed and well-documented during their hospitalization stays. High dimensionality and small sample size becomes a tough combination for traditional correlation-based feature selection methods to perform adequately in discovering the true risk factors. On the other hand, Multiple cause Discovery combined with Structure Learning (McDSL) [6], is a causality discovery method designed to uncover the true causal relations as well as multi-causes structures by effectively removing spurious features on high-dimensional data, which in turn would improve prediction performance. More importantly, the ability to pinpoint the direct causes can aid physicians to design interventions with better efficacy.

In this study, we adopted McDSL algorithm to carry out causal feature selection for the problem of predicting whether an inpatient will develop Stage-3 AKI using clinical information stored in EHR at 24-h prior to the event. To evaluate the effectiveness of selected features, a collection of well-constructed machine learning methods were applied. Prediction accuracy was measured by AUC, F-score, precision and recall based on 10-fold cross-validation results, and compared to predictions made from an ensemble classifier which is built on all the original 891 features.

## Methods

### Data collection

#### Study population

All adult patients (age at visit > 18) hospitalized for at least 2 days at a tertiary care, academic hospital (University of Kansas Health System - KUH) from November 2007 to December 2016 were initially included in the observational cohort study (*n* = 96,590 patients). Given that a patient may have multiple admissions (encounters) of at least 2 days and develop AKI during one but not another, this study is conducted at the encounter level with a total of 179,370 encounters. From these encounters, we excluded those who (a) missed data required for outcome determination, i.e. less than two serum creatinine measurements, and (b) had evidence of moderate or severe kidney dysfunction, i.e. estimated Glomerular Filtration Rate (eGFR) less than 60 mL/min/1.73 m^2^ or abnormal serum creatinine (SCr) level of > 1.3 mg/dL within 24 h of hospital admission. The exclusions finally leave us with 69,698 non-AKI patients and 7259 AKI patients, among whom only 179 progressed to the stage 3. The resulting dataset was highly unbalanced with number of negative observations (non-AKI) more than 389 times the number of positive ones (stage-3 AKI). With such unbalanced dataset, it is very likely for a machine learning model to simply classify everyone as negative case to achieve optimized performance, which will be of little practical use. A common practice for resuming ‘balance’ of a dataset is to match each positive case with one negative case who possesses similar observable characteristics of selection. To favor more on the modifiable features such as medications, laboratory tests and etc. and potentially reduce bias due to confounding, we did the matching upon demographics which are non-modifiable.

Then for each encounter, KUH’s de-identified clinical data repository HERON (Health Enterprise Repository for Ontological Narration) [7] was queried to obtain structured data corresponding to the encounter. HERON integrates data from KUH’s EHR, billing, clinical registries, and national data sources. The structured data extracted included demographic information, admission and discharge dates, medications, laboratory values, comorbidities, and admission diagnosis.

### AKI and Baesline creatinine definition

The staging of AKI is defined by KDIGO criteria [3], as detailed in Table 1. Baseline SCr level is defined as either the last measurement within 2-day time window prior to hospital admission or the first SCr measured after hospital admission. All SCr levels measured between admission and discharge were evaluated to determine the occurrence of inpatient AKI. By matching each of the positive encounters that made to our final analysis cohort, the final study cohort consists of 358 encounters.

**Table 1:** The KDIGO staging system for AKI

AKI Stage	Serum Creatinine (SCr) Criteria
1	Increase > 26.4 μmol/L (0.3 mg/dL) or 1.5–1.9 times baseline
2	Increase 2.0–2.9 times baseline
3	Increase creatinine > 354 μmol/L (4.0 mg/dL) or 3 times baseline

### AKI risk factors

We referred to Matheny et al. [8] for selection of laboratory tests that may represent potential presence of a comorbidity that is correlated with in-hospital AKI. For example, an elevated white blood cell count (WBC) is associated with bacterial infection that may cause AKI. SCr was not included as a predictor as it was used to determine the AKI vs non-AKI encounters. A summary of clinical variables used in building the AKI prediction models is described in Table 2.

**Table 2:** Clinical variables considered in building predictive models for Stage-3 AKI

Feature Category	# of Variable	Details
Demographics	3	Age, gender, race
Vitals	5	BMI, diastolic BP, systolic BP, pulse, temperature
Lab Tests	14	Albumin, ALT, AST, Ammonia, Blood Bilirubin, BUN, Ca, CK-MB, CK, Glucose, Lipase, Platelets, Troponin, WBC
Comorbidities	28	UHC comorbidity
Admission Diagnosis	129	UHC APR-DRG
Medications	482	All medications are mapped to RxNorm ingredient
Medical History	230	ICD9 codes mapped to CCS major diagnoses

For laboratory tests and vitals, only the last recorded value before a prediction point was used and their values were categorized. Values for laboratory tests were categorized as either “present and normal”, “present and abnormal”, or “unknown” according to standard reference ranges. Vitals were discretized into groups as specified in Table 3. Missing values in vitals and lab tests were treated as a separate category called “unknowns”.

**Table 3:** Categories for vital signs

Vitals	Categories
BMI	< 18.5, [18.5–24.9], [25.0–29.9], > 30.0, Unknown
Diastolic BP	< 80, [80–89], [90–99], > 100, Unknown
Systolic BP	< 120, [120–139], [140–159], > 160, Unknown
Pulse	< 50, [50–65], [66–80], [81–100], > 100, Unknown
Temperature	< 95.0, [95.0–97.6], [97.7–99.5], [99.5–104.0], > 104.0, Unknown

Medication variables included inpatient (i.e. dispensed during stay) and outpatient medications (i.e. historical meds). All medication names were normalized by mapping to RxNorm at ingredient level. Comorbidity and admission diagnosis, i.e., all patient refined diagnosis related group (APR-DRG) variables were collected from the University HealthSystem Consortium (UHC) data source in HERON. Patient medical history was captured as major diagnoses (ICD-9 codes grouped according to the Clinical Classifications Software (CCS) diagnosis categories by the Agency for Healthcare Research and Quality). Medical history, medication, comorbidity and admission diagnosis variables took values as “yes” or “no”.

Vitals, labs, medical history and medication variables were time-stamped relative to the admission date, referred here as time-dependent variables. The check point of above categories was a day before Stage-3 AKI breakout. Comorbidities, admission diagnosis, and demographics, in contrast, were presumed to be available at the admission and thus were time-independent.

### Experimental methodology

**Fig. 1:**
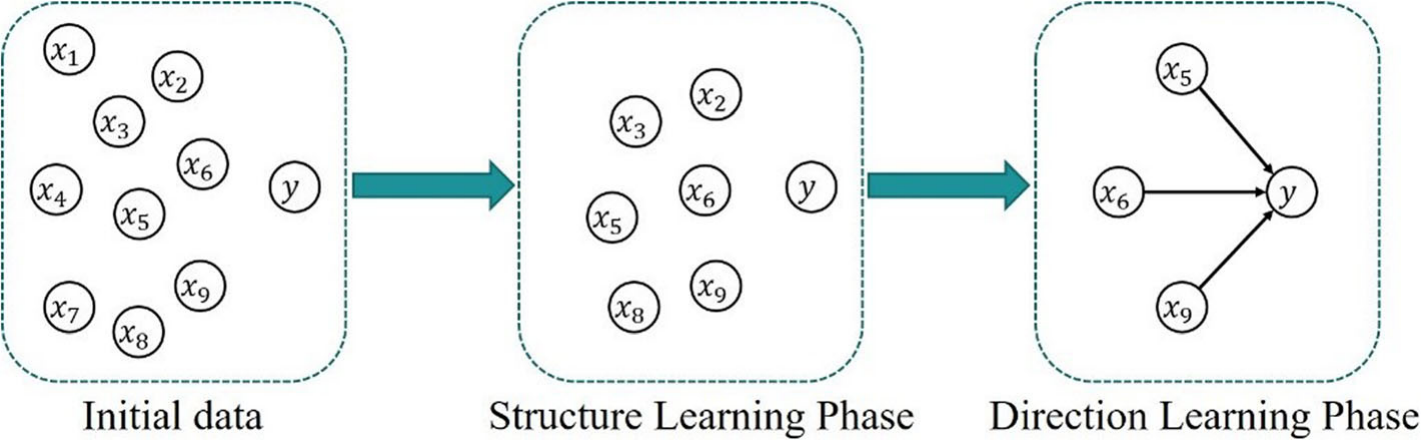
The causal discovery process of McDsL. The structure learning phase is employed for dimensionality reduction, which the potential indirect causal features are deleted. The direction learning phase is proposed for discover many-to-one causalities

The McDSL was adopted to discover the true risk factors of development of Stage-3 AKI out of all 891 features available in EHR. McDSL is a type of generalized causality discovery method suitable for sparse discrete data, which discovers causality in two phases, that is, the structure learning phase and the direction learning phase, as shown in Fig. 1.

In structure learning phase, the d-separation with chi-square test was employed for discovering the Markov blanket which includes potential causes and their effects on the target of interest. In direction learning phase, the direct causes is discovered from the potential ones by incorporating ANM with a conversion method that converts several features into one, as ANM is a nonlinear functional causal model (FCM) yet only works on one-to-one causal structure (accuracies of different datasets: 89~ 97% [9]). An FCM represents the effect variable *y* as a function of the direct causes *x* and some noise *N*, i.e. *y* = *f*(*x*, *N*), where *N* is independent of *x*, and it is violated for the reverse direction. McDSL develop the FCM for discovering the many-to-one causal structure. It represents the effect variable *y* as a function of the a converted direct causes $\overline{x}=g\left({x}_1,{x}_2,\dots \right)$ and some noise *N*, i.e.$y=f\left(\overline{x},N\right)$, where *N* is independent of $\overline{x}$, and it is violated for the reverse direction. The many-to-one causal structure is tenable if and only if the unique set of all causes had discovered, which is denoted as {*x*_1_, *x*_2_, …} → *y*.

Table 4 shows the converted process of $\overline{x}=g\left({x}_5,{x}_6,{x}_9\right)$ in Fig. 1. Where ${v}_i^j$ means the *j*-th state of features *x*_*i*_, *m*_*i*_ is the scale of states of *x*_*i*_, $\overline{x}$ is the converted feature, and its states are the combinations of *x*_5_, *x*_6_ and *x*_9_.

**Table 4:** The converted model of two features

	*x*_5_	*x*_6_	*x*_9_	$\overline{x}$
states	${v}_5^1$	${v}_6^1$	${v}_9^1$	1
	⋮	⋮	⋮	⋮
	${v}_5^1$	${v}_6^1$	${v}_9^{m_9}$	*m*_9_
	${v}_5^1$	${v}_6^2$	${v}_9^1$	*m*_9_ + 1
	⋮	⋮	⋮	⋮
	${v}_5^{m_5}$	${v}_6^{m_6}$	${v}_9^{m_9}$	*m*_5_ *m*_6_ *m*_9_

The effectiveness of McDSL has been recognized in synthetic data as well as several application areas, such as stock risk prediction [6] and software projective risk analysis [10].

To assess the explanatory and predictive power of the risk factors being extracted, prediction accuracies were examined over a variety of machine learning models which were built using only the causal features. K-nearest neighbor (KNN) [11], decision trees (DT) [12], backpropagation neural network (BPNN) [13], random forest (RF) [14] and an ensemble classifier (EC) [15] were selected for the task as they have been well-established in the literature of relevant context and represent predictive models assuming different underlying structures. KNN is a non-parametric statistical model that can learn both linear and non-linear relationships but with less stringent assumptions than any conventional regression models. Decision tree and BPNN are both rule-based classifiers, while the former can be human-interpretable but the latter not so much. However, BPNN has been shown, theoretically or experimentally, to be competitive advantageous on performing prediction tasks if efficient predictors are used. Random forest and the ensemble classifier are two ensemble learning methods. An ensemble method typically obtains better results than component classifiers by joining multiple classification methods together [16]. But it is not always the case if the component classifiers agree most of the time by capturing similar signals.

## Results

### Causal risk factor discovery

McDSL discovered four risk factors of Stage-3 AKI from those 891 features, all of which are medications and mostly pertinent to gastrointestinal system. Specifically, they are 1) Sennosides, a laxative to treat constipation and empty the large intestine before surgery; 2) 1,2,6-hexanetriol, a moisturizing agent for various creams; 3) Famotidine, a medication used in the treatment of peptic ulcer disease and gastroesophageal reflux disease, and 4) Benzimidazole, a drug class includes many anthelmintic drugs used for the treatment of a variety of parasitic worm infestations.

To verify the causalities, the odd ratios (OR) of all 15 combinations of the four risk factors are presented in Table 5 as well as individually, with 1 indicating usage of this medication and 0 otherwise. The OR results show that the combinations of discovered four medications are correlated to Stage-3 AKI. Moreover, inpatients were given medications at least one day earlier than the onset of the disease. Therefore, those previous features are the causes of Stage-3 AKI risk in the temporal sequence.

**Table 5:** OR and 95% CI of combinations of discovered risk factors

Combinations of risk factors	Risk factors				Odd Ratio [95% CI]
	#1	#2	#3	#4	
CoRF1	1	0	0	0	1.24 [0.69, 2.23]
CoRF2	0	1	0	0	1.47 [0.92, 2.34]
CoRF3	0	0	1	0	0.64 [0.26, 1.56]
CoRF4	0	0	0	1	0.66 [0.37, 1.19]
CoRF5	1	0	0	1	0.62 [0.30, 1.25]
CoRF6	1	0	1	0	0.48 [0.18, 1.29]
CoRF7	1	1	0	0	1.56 [0.90, 2.70]
CoRF8	0	1	0	1	1.48 [0.97, 2.26]
CoRF9	0	1	1	0	1.24 [0.67, 2.28]
CoRF10	0	0	1	1	0.30 [0.04, 2.12]
CoRF11	1	0	1	1	0.28 [0.04, 1.97]
CoRF12	1	1	0	1	0.94 [0.58, 1.53]
CoRF13	1	1	1	0	0.92 [0.52, 1.62]
CoRF14	0	1	1	1	1.20 [0.53, 2.72]
CoRF15	1	1	1	1	1.18 [0.66, 2.12]

### Stage-3 AKI risk prediction

**Fig. 2:**
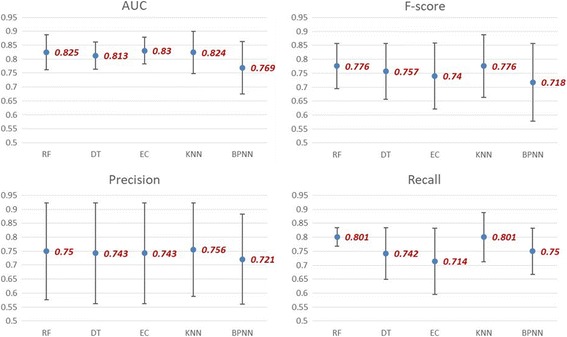
The prediction results of McDSL with five machine learning methods. AUC, F-score, Precision, Recall, and 95% confidence intervals obtained using different machine learning methods, with 10-fold cross-validation, for predicting Stage-3 AKI during hospital stay

Using only the feature set suggested by MsDCL, the five machine learning models aforementioned were built for predicting Stage-3 AKI with 10-fold cross-validation and their predictive accuracy were measured by AUC, precision, recall and F-score. As displayed in Fig. 2.

As demonstrated in Table 6, the further comparisons are made against the McDSL combined with a review research of AKI (McDSL + PLoS one) [17] and Logistic regression (LR) [18]. The experimental result shows that McDSL has discovered the key features for predicting the Stage-3 AKI risk.

**Table 6:** The comparison of different feature selected features

Models (# of variable)	AUC	F-score	Recall	Precision
McDSL (4)	0.812	0.753	0.761	0.743
McDSL + PLoS one (6)	0.814	0.748	0.744	0.738
LR (88)	0.837	0.775	0.810	0.734

## Discussion

The AKI is a common clinical event with high morbidity and mortality, and Stage-3 AKI is the worst. Discovery the direct causes of Stage-3 AKI from EHR is valuable for promoting for clinical research. This study adopts McDSL, which is a functional causal model, for discovering the causes of Stage-3 AKI risk. Four risk features are inferred as the causes of Stage-3 AKI risk, and those causalities are verified by OR. The subsequent experiments show that those causes are the key features to predict the Stage-3 AKI risk.

### Causes of Stage-3 AKI risk

Causal discovery from observed data is a hotspot of big data research [19, 20] which can avoid the ethics risk and reduce the cost of intervention experiment. EHR is a kind of structured observed data, and it was became the focus of machine learning research [21]. The data of Stage-3 AKI is sparse and high dimensional, and it is unfeasible for correlation-based feature selection or modeling method. Therefore, McDSL is employed to discover the causes of Stage-3 AKI risks with two phases: dimensionality reduction and causal direction inferring.

The causalities between four medications and the Stage-3 AKI risks has discovered by McDSL. To verify the accuracy of discovered causalities, the OR of all combinations of four features has calculated, as shown in Table 5. The experimental results show that seven groups of OR are greater than 1. The ORs of Sennosides (CoRF1, OR 1.24) and 1,2,6-hexanetriol (CoRF2, OR 1.47) as well as most of the combinations involving these two medications (e.g. 1,2,6-hexanetriol and Benzimidazole (CoRF8, OR 1.48); 1,2,6-hexanetriol, Famotidine and Benzimidazole (CoRF14, OR 1.20)) can be observed to be greater than 1. The OR results suggest combinatorial effects of the four McDSL selected features certainly exist. Moreover, the check point of medication variables are at least a day earlier than breakout of Stage-3 AKI. Therefore, these four features are the causes of Stage-3 AKI risk.

### Advantage of McDSL for Stage-3 AKI detection

To detect the risk of Stage-3 AKI earlier, it is necessary to discover the accurate risk features. Five machine learning models were employed to predict Stage-3 AKI risk with 10-fold cross-validation. The AUC of all the models ranges between 0.769 and 0.830, among which four models (RF, DT, KNN and EC) could even achieve AUC greater than 0.810. Precision of all the models ranges from 0.721 to 0.756, indicating that the models, which solely relies on the four selected features, could successfully identify the Stage-3 AKI patients for more than 72% of the time among all the true patients. Recall, taking values between 0.714 and 0.801, suggests that all of the models are capable of returning more relevant results than irrelevant ones. The F-score, a balanced performance metric of precision and recall, ranges from 0.718 to 0.776, with three models (RF, DT and KNN) all scored above 0.750. In view of all the four accuracy measurements under consideration, the predictive power of the four selected features are fairly robust across various modeling techniques.

In addition, a comparison of different feature selected features was presented to estimate the advantage of McDSL. Ohnuma and Uchino had proposed a systematic review of prediction models for mortality of patients with acute kidney injury, and shows that age and gender were the most common risk features of AKI. The AUC of this combination is close to McDSL (+ 0.2%). Moreover, LR is a parametric statistical models and frequently-used prediction model for EHR, and it selected 88 risk features. Although LR selected much more risk features than McDSL, the improvement is very small (+ 3%). Therefore, the selected four features are the key risk features of prediction Stage-3 AKI.

## Conclusions

This study extended the application of McDSL to a new domain, that is, to discover true risk factors of Stage-3 AKI from EHR, which has become an emerging valuable source for conducting large-scale clinical research studies. Four modifiable features have been identified, which suggests prospective practical use in managing and treating Stage-3 AKI patients during their hospital stay. Classification accuracy can be preserved and even improved by learning from the features selected by McDSL. Based on our experimental results, it is promising to further extend such causal feature selection methodology as McDSL to discovering true risk factors associated with stage-1 and stage-2 AKI events.

## Abbreviations

ANM: Additive noise model
EHR: Electronic health record
McDSL: Multiple cause Discovery combined with Structure Learning
